# IgGs from patients with amyotrophic lateral sclerosis and diabetes target Ca_V_α_2_δ1 subunits impairing islet cell function and survival

**DOI:** 10.1073/pnas.1911956116

**Published:** 2019-12-11

**Authors:** Yue Shi, Kyoung Sun Park, Seung Hyun Kim, Jia Yu, Kaixuan Zhao, Lina Yu, Ki Wook Oh, Kayoung Lee, Jaeyoon Kim, Kanchan Chaggar, Yuxin Li, Annette C. Dolphin, William A. Catterall, Sung Ho Ryu, Shao-Nian Yang, Per-Olof Berggren

**Affiliations:** ^a^The Rolf Luft Research Center for Diabetes and Endocrinology, Karolinska Institutet, Karolinska University Hospital L1, SE-171 76 Stockholm, Sweden;; ^b^Division of Integrative Biosciences and Biotechnology, Pohang University of Science and Technology, Pohang 37673, Korea;; ^c^Department of Neurology, Hanyang University Hospital, Seoul 04763, Korea;; ^d^Department of Neuroscience, Physiology and Pharmacology, University College London, London WC1E 6BT, United Kingdom;; ^e^National Engineering Laboratory for Druggable Gene and Protein Screening, Northeast Normal University, Changchun 130024, China;; ^f^Department of Pharmacology, School of Medicine, University of Washington, Seattle, WA 98195-7280;; ^g^Lee Kong Chian School of Medicine, Nanyang Technological University, Singapore 637553;; ^h^Diabetes Research Institute, Miller School of Medicine, University of Miami, Miami, FL 33136

**Keywords:** amyotrophic lateral sclerosis, calcium channel, cytosolic free Ca^2+^ concentration, diabetes, immunoglobulin

## Abstract

Patients with amyotrophic lateral sclerosis (ALS) often show hallmarks of type 2 diabetes mellitus (T2DM). However, the causal link between ALS and T2DM has remained a mystery. We now demonstrate that 60% of ALS patients with T2DM (ALS-T2DM) have sera that exaggerated K^+^-induced increases in cytosolic free Ca^2+^ concentration ([Ca^2+^]_i_) in mouse islet cells. The effect was attributed to the presence of pathogenic immunoglobulin Gs (IgGs) in ALS-T2DM sera. The pathogenic IgGs immunocaptured the voltage-dependent Ca^2+^ (Ca_V_) channel subunit Ca_V_α_2_δ1 in the plasma membrane enhancing Ca_V_1 channel-mediated Ca^2+^ influx and [Ca^2+^]_i_, resulting in impaired mitochondrial function. Consequently, impairments in [Ca^2+^]_i_ dynamics, insulin secretion, and cell viability occurred. These data reveal that patients with ALS-T2DM carry cytotoxic ALS-T2DM-IgG autoantibodies that serve as a causal link between ALS and T2DM by immunoattacking Ca_V_α_2_δ1 subunits. Our findings may lay the foundation for a pharmacological treatment strategy for patients suffering from a combination of these diseases.

## Significance

We provide evidence of a mechanistic link between ALS and T2DM. Our data show that a subgroup of ALS-T2DM patients have sera that enhance Ca_V_1 channel-mediated Ca^2+^ influx and exaggerate [Ca^2+^]_i_. These effects occur because the sera accommodate cytotoxic IgG autoantibodies that immunocapture Ca_V_α_2_δ1 subunits. As a consequence, impairments in [Ca^2+^]_i_ dynamics, mitochondrial function, insulin secretion, and cell viability appear. We could clarify not only the identity of this serum factor but also the molecular mechanisms underlying its effects on the islet cells. Our findings may lay the foundation for a treatment strategy for this complex and severe group of diabetic patients.

Patients with amyotrophic lateral sclerosis (ALS) show progressive dysfunction and degeneration of motor neurons in the brainstem and spinal cord (1). There are no effective therapeutics available for ALS (1). Clinically, ALS patients manifest advanced muscular weakness and paralysis and die within 2 to 5 y from the onset of the disease (1). Although the exact pathogenic mechanisms of ALS are not clarified, dysregulation of voltage-dependent Ca^2+^ (Ca_V_) channels, cytosolic free Ca^2+^ concentration ([Ca^2+^]_i_), and synaptic plasticity induced by altered humoral immunity has been proposed to participate in the development of the disease (2, 3). Exposure to purified immunoglobulin G (IgG) from the serum of ALS patients produced ultrastructural abnormalities with Ca^2+^ accumulation in and increased transmitter release from rodent motor neurons (4). It has long been recognized that intracellular accumulation of Ca^2+^ is cytotoxic and causes mitochondrial dysfunction, free radical damage, and Ca^2+^-dependent cell death (5–7).

A series of neurodegenerative diseases, like ALS, is known to be associated with hallmarks of type 2 diabetes mellitus (T2DM), such as impaired glucose homeostasis, but their causal links are not known (8–10). Blood glucose homeostasis is under strict control of hormone release from pancreatic islet cells. T2DM and its characteristic symptom hyperglycemia occur in people with inadequate islet cell mass and function. Islet cells and neurons share a series of physiological and pathological mechanisms, such as Ca^2+^-dependent exocytosis and Ca^2+^-triggered cell death, for their function/dysfunction and survival/death (11–14). Therefore, the present study hypothesized that IgG from ALS patients with T2DM (ALS-T2DM) may recognize similar targets in islet cells, as revealed in motor neurons, and thereby impair islet cell function and viability by disturbing Ca^2+^ signaling. Indeed, the present study reveals that a subgroup of ALS-T2DM patients has sera that enhance K^+^-induced [Ca^2+^]_i_ responses in islet cells via cytotoxic IgGs. Moreover, it demonstrates that ALS-T2DM-IgGs immunocapture Ca_V_α_2_δ1 subunits and thereby enhance Ca_V_1 channel-mediated Ca^2+^ influx, resulting in altered [Ca^2+^]_i_ dynamics and, consequently, impaired mitochondrial function, insulin secretion, and cell viability.

## Results

### A Subgroup of ALS-T2DM Patients Has Sera That Exaggerate K^+^-Induced [Ca^2+^]_i_ Responses in Mouse Islet Cells.

**Fig. 1.**
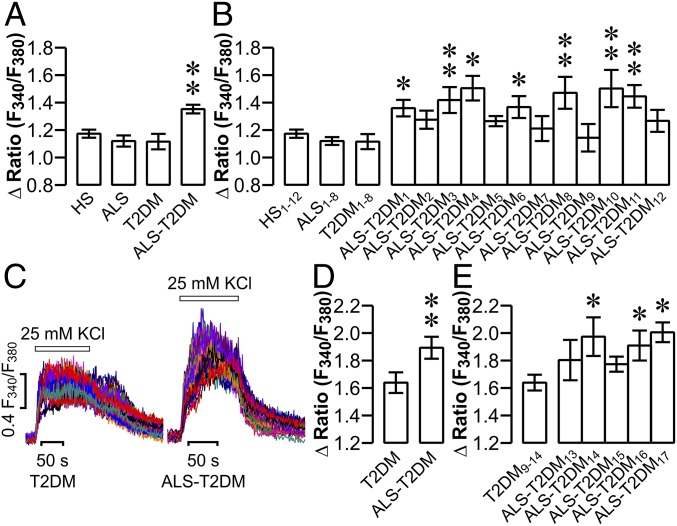
Effects of ALS-T2DM sera on KCl-induced [Ca^2+^]_i_ responses in mouse islet cells. (*A*) Δ Fura-2 F340/F380 ratios showing average net increases in [Ca^2+^]_i_ evoked by K^+^ depolarization in groups subjected to 10 h of treatment with ALS-T2DM (*n* = 12), HS (*n* = 12), ALS (*n* = 8), or T2DM sera (*n* = 8) obtained from the first batch of patients. (*B*) Δ Fura-2 F340/F380 ratios illustrating net increases in [Ca^2+^]_i_ induced by K^+^ stimulation in cells treated for 10 h with 12 individual ALS-T2DM sera as well as 12 HS, 8 ALS ,and 8 T2DM sera collected from the first batch of patients. (*C*) Example recordings of Fura-2 F340/F380 ratios showing [Ca^2+^]_i_ responses to 25 mM KCl in cells following 10 h of incubation with ALS-T2DM serum (*Right*) or T2DM serum (*Left*). (*D*) Δ Fura-2 F340/F380 ratios illustrating mean net increases in [Ca^2+^]_i_ induced by K^+^ stimulation in groups treated for 10 h with ALS-T2DM (*n* = 5) and T2DM sera (*n* = 6) donated by the second batch of patients. (*E*) Δ Fura-2 F340/F380 ratios showing net increases in [Ca^2+^]_i_ evoked by K^+^ depolarization in cells treated for 10 h with 5 individual ALS-T2DM and 6 T2DM sera obtained from the second batch of patients. Ten out of 17 positive ALS-T2DM sera significantly enhance KCl-induced [Ca^2+^]_i_ responses in comparison to HS, ALS, and T2DM sera. *P < 0.05 and **P < 0.01 vs. T2DM, ALS, or HS group.

ALS patient sera accommodate altered humoral immunity that results in pathological exaggeration of voltage-dependent Ca^2+^ entry and [Ca^2+^]_i_ in motor neurons, thereby damaging these cells in a Ca^2+^-dependent manner (2–4). This prompted us to explore if ALS-T2DM serum drives similar pathological events in mouse islet cells. To implement such an exploration, we collected 4 types of sera from healthy human subjects (HSs) and patients with ALS, T2DM, and ALS-T2DM (*SI Appendix*, Table S1). During the course of the present study, 2 separate batches of sera were collected. The first batch of sera was obtained from 12 patients with ALS-T2DM as well as 12 HSs, 8 patients with ALS, and 8 patients with T2DM as controls (*SI Appendix*, Table S1). With each individual serum we treated dissociated islet cells and conducted high-throughput measurements of K^+^ depolarization-induced [Ca^2+^]_i_ responses. General analysis of pooled data revealed that average [Ca^2+^]_i_ response to stimulation with 25 mM KCl in the ALS-T2DM serum-treated group was significantly greater than that in groups subjected to treatment with HS, ALS, or T2DM sera (Fig. 1*A*). Furthermore, the 3 latter groups did not significantly differ in this parameter (Fig. 1*A*). Interestingly, thorough analysis of individual data showed that 7 out of 12 ALS-T2DM sera produced significant increases in [Ca^2+^]_i_ in response to 25 mM KCl, whereas the rest did not, in comparison to HS, ALS, and T2DM sera (Fig. 1 *B* and *C*). To corroborate the results obtained with the first batch of sera, we repeated [Ca^2+^]_i_ measurements with the second batch of sera donated by 5 patients with ALS-T2DM and 6 T2MD patients (*SI Appendix*, Table S1 and Fig. 1 *D* and *E*). Consistent with the first batch of sera, the ALS-T2DM group displayed a significant increase in mean [Ca^2+^]_i_ response to KCl depolarization compared to the T2DM group, and 3 out of 5 ALS-T2DM sera gave rise to significant elevations in K^+^-induced [Ca^2+^]_i_ responses in comparison to T2DM sera (Fig. 1 *D* and *E*). Moreover, the specific Ca_V_1 channel blocker nifedipine almost completely ablated K^+^-induced [Ca^2+^]_i_ responses in mouse islet cells (*SI Appendix*, Fig. S1). In addition, there was no significant difference in basal [Ca^2+^]_i_ between T2DM and ALS-T2DM groups (Fura-2 F340/F380 ratio for the T2DM group of 0.537 ± 0.028 vs. Fura-2 F340/F380 ratio for the ALS-T2DM group of 0.541 ± 0.017, *P* > 0.05). Taken together, 60% of ALS-T2DM patients have sera that authentically exaggerate K^+^-induced [Ca^2+^]_i_ responses in islet cells. These ALS-T2DM sera were defined as positive ALS-T2DM sera and randomly chosen for subsequent experiments.

### Pathogenic IgGs Present in Positive ALS-T2DM Sera Enhance K^+^-Induced [Ca^2+^]_i_ Responses in Mouse Islet Cells.

**Fig. 2.**
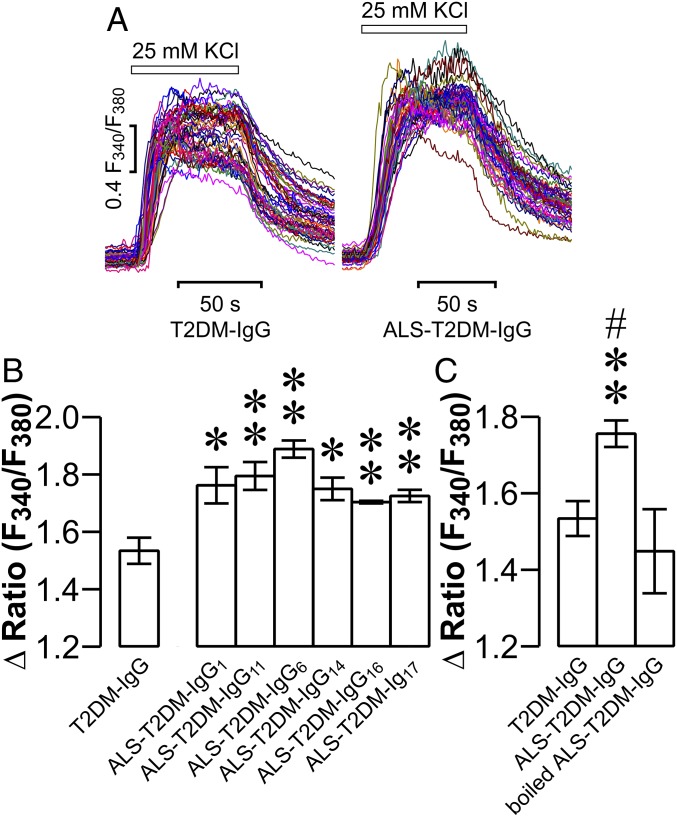
Effects of IgGs purified from positive ALS-T2DM sera on KCl-induced [Ca^2+^]_i_ responses in mouse islet cells. (*A*) Representative [Ca^2+^]_i_ responses to 25 mM KCl in islet cells following exposure to T2DM-IgG (*Left*) and ALS-T2DM-IgG (*Right*). (*B*) Δ Fura-2 F340/F380 ratios showing net increases in [Ca^2+^]_i_ evoked by 25 mM KCl in islet cells treated with IgGs purified from 8 T2DM sera or 6 positive ALS-T2DM sera. *P < 0.05 and **P < 0.01 vs. the T2DM-IgG group. (*C*) Δ Fura-2 F340/F380 ratios showing net increases in [Ca^2+^]_i_ in islet cells treated with T2DM-IgG, ALS-T2DM-IgG, or boiled ALS-T2DM-IgG. Experiments were done with 8 T2DM-IgGs, 6 ALS-T2DM-IgGs, or 6 boiled ALS-T2DM-IgGs. **P < 0.01 vs. the T2DM-IgG group and ^#^*P* < 0.05 vs. the boiled ALS-T2DM-IgG group.

It has been demonstrated that pathogenic IgGs reside in sera of ALS patients and account for a great deal of Ca^2+^-dependent destruction of motor neurons and skeletal muscle cells (4, 15–18). This raised the question of whether IgGs in positive ALS-T2DM sera (ALS-T2DM-IgGs) also serve as molecular pathogenic factors to impair islet cell function and survival by perturbing [Ca^2+^]_i_ homeostasis. To tackle this question, we purified IgGs from positive ALS-T2DM sera and T2DM sera. Thereafter, we measured the effects of these purified IgGs on K^+^-induced [Ca^2+^]_i_ responses in islet cells. Incubation with these individual ALS-T2DM-IgGs induced significantly stronger [Ca^2+^]_i_ responses to 25 mM KCl in comparison to exposure to IgGs from T2DM sera (T2DM-IgGs) in mouse islet cells (Fig. 2 *A* and *B*). Furthermore, the effect of ALS-T2DM-IgGs was lost when boiled (Fig. 2*C*). Cells exposed to either boiled ALS-T2DM-IgGs or T2DM-IgGs responded similarly to KCl stimulation with regard to increases in [Ca^2+^]_i_ (Fig. 2*C*). These data demonstrate that ALS-T2DM-IgG in ALS-T2DM sera enhances K^+^-induced [Ca^2+^]_i_ responses.

### Positive ALS-T2DM Sera Up-regulate Ca_V_1 Channels through Direct Interaction with Ca_V_α_2_δ1 Subunits in Mouse Islet Cells.

Autoantibodies against Ca_V_1.1, Ca_V_2.1, and Ca_V_2.2 subunits have been demonstrated to be present in ALS patients (15, 16). Importantly, these autoantibodies enhance Ca^2+^ conductivity of these Ca^2+^-conducting pores, resulting in excessively high [Ca^2+^]_i_ and, consequently, Ca^2+^-dependent cytotoxicity in skeletal muscle cells and neurons (4, 15–19). Of particular importance is that selective Ca_V_1.1, Ca_V_2.1, and Ca_V_2.2 channel blockers substantially improve defects in neuromuscular activity and viability induced by IgGs from ALS patients (2, 18, 20–23). Our finding that both ALS-T2DM serum and ALS-T2DM-IgG promote K^+^-evoked [Ca^2+^]_i_ responses suggests that Ca_V_1.2 channels might serve as downstream targets of ALS-T2DM serum and ALS-T2DM-IgG. It is well known that depolarization-evoked [Ca^2+^]_i_ responses in mouse islet cells primarily result from Ca^2+^ influx through Cav1.2 channels (24).

**Fig. 3.**
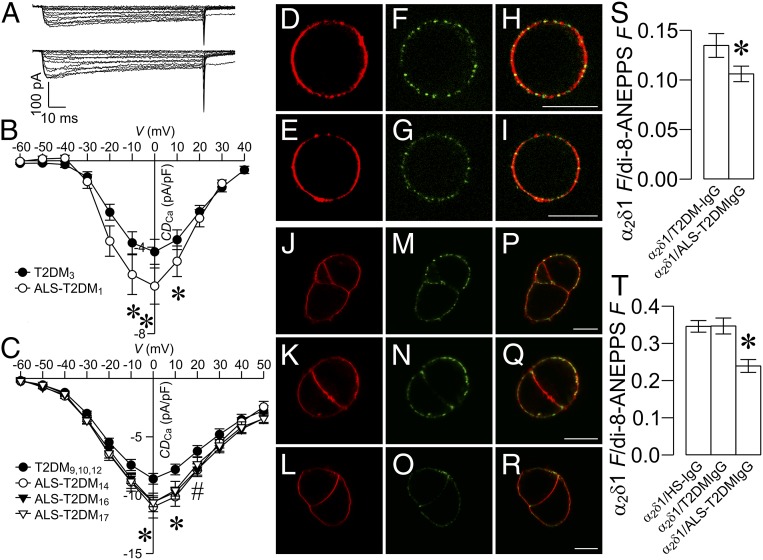
Effects of positive ALS-T2DM sera on whole-cell Ca_V_ currents in mouse islet cells and influences of ALS-T2DM-IgGs on Ca_V_α_2_δ1 immunofluorescence in the plasma membrane of living mouse islet cells and tsA-201 cells stably expressing Ca_V_α_2_δ1 subunits. (*A*) Representative whole-cell Ca^2+^ currents in islet β cells incubated with T2DM serum (*Upper*) or ALS-T2DM serum (*Lower*) for 10 h. (*B* and *C*) Whole-cell current–voltage relationships of Ca_V_ channels in cells treated with the first batch (*B*) of one T2DM or one ALS-T2DM serum and with the second batch (*C*) of 3 T2DM or 3 ALS-T2DM sera. *P < 0.05 for the ALS-T2DM_1_ group vs. the T2DM_3_ group in *B*; *P < 0.05 for the ALS-T2DM_14_, ALS-T2DM_16_, or ALS-T2DM_17_ group vs. the T2DM_9,10,12_ group and ^#^*P* < 0.05 for the ALS-T2DM_14_ or ALS-T2DM_16_ group vs. the T2DM_9,10,12_ group in (*C*). *CD*_Ca_, Ca^2+^ current density. (*D*–*R*) Representative di-8-ANEPPS fluorescence (*D*, *E*, *J*, *K*, and *L*), Ca_V_α_2_δ1 immunofluorescence (*F*, *G*, *M*, *N*, and *O*), and their overlay images (*H*, *I*, *P*, *Q*, and *R*) of living mouse islet cells (*D*–*I*) and tsA-201 cells stably expressing Ca_V_α_2_δ1 subunits (*J*–*R*) incubated with rabbit polyclonal anti-Ca_V_α_2_δ1 antibodies in the presence of HS-IgGs (*J*, *M*, and *P*), T2DM-IgGs (*D*, *F*, *H*, *K*, *N*, and *Q*), and ALS-T2DM-IgGs (*E*, *G*, *I, L*, *O*, and *R*). (Scale bar, 10 μm.) (*S* and *T*) Mean Ca_V_α_2_δ1 immunofluorescence/di-8-ANEPPS fluorescence ratio in living mouse islet cells (*S*) and tsA-201 cells stably expressing Ca_V_α_2_δ1 subunits (*T*) incubated with Ca_V_α_2_δ1 antibodies plus HS-IgGs (Ca_V_α_2_δ1/HS-IgG), T2DM-IgGs (Ca_V_α_2_δ1/T2DM-IgG), and ALS-T2DM-IgGs (Ca_V_α_2_δ1/ALS-T2DM-IgG). Experiments were done with 3 T2DM and 3 ALS-T2DM sera. *P < 0.05 vs. the Ca_V_α_2_δ1/HS-IgG group or Ca_V_α_2_δ1/T2DM-IgG group.

To clarify if ALS-T2DM serum affects β cell Ca_V_ channels, we examined the effect of positive ALS-T2DM serum on β cell Ca_V_ channel currents. Indeed, whole-cell patch-clamp analysis revealed that treatment with individual positive ALS-T2DM sera obtained in the first and second batches significantly elevated whole-cell Ca_V_ channel currents in mouse β cells, as manifested by representative whole-cell Ca^2+^ current traces and average Ca_V_ channel current density, in comparison to T2DM serum exposure (Fig. 3 *A*–*C*). These data verify that ALS-T2DM serum up-regulates Ca_V_1 channels, causing pathologically exaggerated [Ca^2+^]_i_ responses.

The up-regulation of Ca_V_1 channels by ALS-T2DM serum raises the possibility that IgGs in ALS-T2DM serum may target Ca_V_ channel subunits in β cells. We chose the most important pore-forming subunit Ca_V_1.2 as a starting point. Immunoprecipitation assays followed by immunoblot analysis showed that antibodies against Ca_V_1.2 subunits efficiently pulled down Ca_V_1.2 subunits from the membrane fraction of insulin-secreting RINm5f cells (*SI Appendix*, Fig. S2*A*). However, neither ALS-T2DM-IgGs nor T2DM-IgGs could recognize the immunoprecipitated Ca_V_1.2 subunits under denaturing or renaturing conditions (*SI Appendix*, Fig. S2 *B* and *C*). Furthermore, both ALS-T2DM-IgGs and T2DM-IgGs could not specifically fish out Ca_V_1.2 subunits and additional proteins (*SI Appendix*, Fig. S2 *D* and *E*). These results show that ALS-T2DM-IgGs could not strongly bind to immunoprecipitated Ca_V_1.2 subunits under such experimental conditions.

The Ca_V_α_2_δ1 subunit, an important constituent of Ca_V_ channel complexes, including the β cell Ca_V_1.2 channel complex, is critical for the surface expression of functional Ca_V_ channels (12, 25–27). Moreover, the entire Ca_V_α_2_δ1 is exposed extracellularly (28). Among all β cell Ca_V_1.2 channel components, they have the highest likelihood of serving as targets for factors, such as ALS-T2DM-IgGs, of positive ALS-T2DM sera. In addition, polyclonal anti-Ca_V_α_2_δ1 antibodies selectively recognize extracellular Ca_V_α_2_δ1 subunits associated with the plasma membrane of living cells bathed in a physiological solution (28, 29). This prompted us to clarify if ALS-T2DM-IgGs interacts with Ca_V_α_2_δ1 subunits under physiological conditions by using anti-Ca_V_α_2_δ1 antibodies. We carried out 4-(2-[6-(Dioctylamino)-2-naphthalenyl]ethenyl)-1-(3-sulfopropyl)pyridinium inner salt (di-8-ANEPPS) labeling of the plasma membrane and immunofluorescence staining of Ca_V_α_2_δ1 subunits in intact living mouse islet cells and tsA-201 cells stably expressing Ca_V_α_2_δ1 subunits coincubated with antibodies against Ca_V_α_2_δ1 subunits and ALS-T2DM-IgGs. Ca_V_α_2_δ1-specific immunofluorescence was clearly localized in the di-8-ANEPPS-labeled plasma membrane (Fig. 3 *D*–*R* and *SI Appendix*, Fig. S3). Interestingly, ALS-T2DM-IgGs effectively competed with the anti-Ca_V_α_2_δ1 antibodies for the extracellular Ca_V_α_2_δ1 subunits, resulting in a significant reduction in the immunofluorescence intensity of the anti-Ca_V_α_2_δ1 antibodies in comparison to HS-IgGs or T2DM-IgGs (Fig. 3 *S* and *T*). This verifies that ALS-T2DM-IgGs are capable of directly interacting with Ca_V_α_2_δ1 subunits in living cells in the absence of interferences from detergents, high ionic strengths, and substantial rinsing, which are unavoidable in immunoprecipitation and immunoblot analyses of association between ALS-T2DM-IgGs and Ca_V_1.2 subunits.

### Positive ALS-T2DM Sera Interfere with Mitochondrial Function in Mouse Islet Cells.

**Fig. 4.**
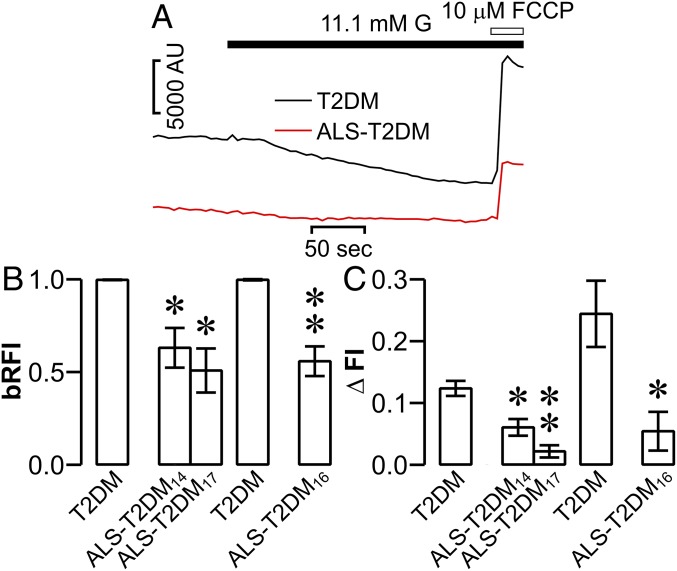
Effects of positive ALS-T2DM sera on mouse islet cell mitochondrial membrane potential. (*A*) Sample rhodamine 123 fluorescence traces registered in a T2DM serum-treated cell (black trace) and in a cell exposed to ALS-T2DM serum (red trace) following glucose (G) and FCCP stimulation. FCCP: carbonyl cyanide *p*-trifluoromethoxyphenylhydrazone, AU: arbitrary units. (*B*) Basal relative rhodamine 123 fluorescence intensities (bRFI) in cells incubated with T2DM, ALS-T2DM_14_, and ALS-T2DM_17_ sera for 24 h as well as T2DM and ALS-T2DM_16_ sera for 12 h. (*C*) Net changes in glucose-induced quenching of rhodamine 123 (ΔFI) in cells treated with T2DM, ALS-T2DM_14_, and ALS-T2DM_17_ sera for 24 h as well as T2DM and ALS-T2DM_16_ sera for 12 h. Experiments were done with 2 T2DM and 3 ALS-T2DM sera.*P < 0.05 and **P < 0.01 vs. the T2DM group.

Translation of an excessive elevation of [Ca^2+^]_i_ into mitochondrial Ca^2+^ overload results in mitochondrial membrane depolarization, concomitant mitochondrial dysfunction, and eventual apoptosis, thus playing an important role in driving Ca^2+^-dependent cell death (30). This made us wonder whether such a mitochondrial mechanism is able to convert the ALS-T2DM serum-induced exaggeration of [Ca^2+^]_i_ to mitochondrial dysfunction in mouse islet cells. Therefore, we measured mitochondrial membrane potential in mouse islet cells using rhodamine 123. As shown in Fig. 4 *A*–*C*, ALS-T2DM serum-treated cells displayed a significant decrease not only in basal fluorescence intensity of rhodamine 123 (Fig. 4 *A* and *B*) but also in the glucose-induced quenching of rhodamine 123 (Fig. 4 *A* and *C*), compared to those cells incubated with T2DM serum. The effects are attributed to reduced amounts of rhodamine 123 loaded into mitochondria due to less negative mitochondrial membrane potential, i.e., mitochondrial dysfunction, induced by ALS-T2DM serum treatment (31). Our results demonstrate that mouse islet cells insulted by exaggerated [Ca^2+^]_i_ resulting from exposure to positive ALS-T2DM sera undergo mitochondrial dysfunction and suggest that ALS-T2DM serum-induced mitochondrial dysfunction is most likely to drive islet cell death.

### Positive ALS-T2DM Sera Impair [Ca^2+^]_i_ Dynamics and Insulin Secretion in Mouse Islets.

Normal glucose homeostasis critically relies on adequately functioning β cells (32). The function of β cells is under the control of exquisitely fine-tuned [Ca^2+^]_i_ dynamics that serves as fingerprints for β cell well-being (12, 13, 24, 33, 34). This made us wonder if ALS-T2DM serum drives disorganized [Ca^2+^]_i_ dynamics and impaired insulin secretion in islets, thereby accounting for aberrant glucose homeostasis often observed in ALS patients.

**Fig. 5.**
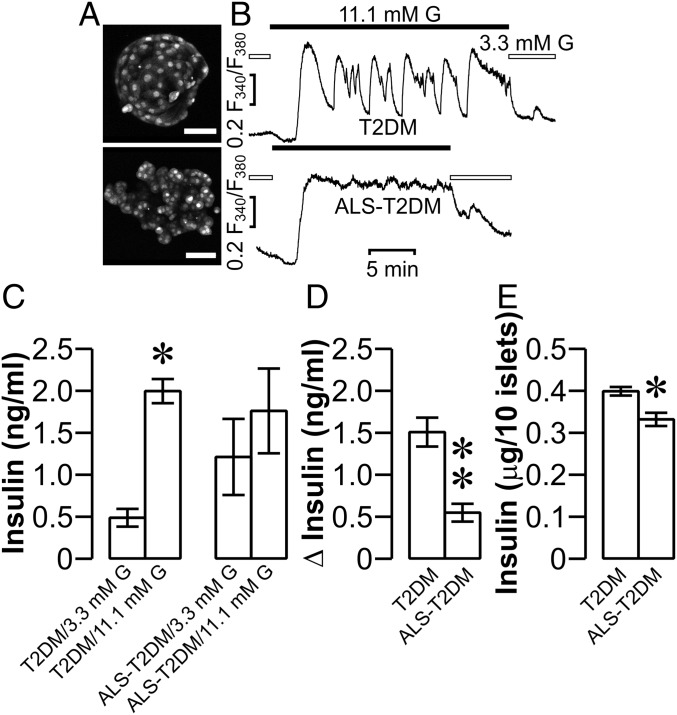
Effects of positive ALS-T2DM sera on mouse islet [Ca^2+^]_i_ dynamics and insulin secretion. (*A*) Sample photomicrographs of Fura-2-loaded mouse islets following 10 h of treatment with T2DM (*Upper*) and ALS-T2DM serum (*Lower*). (Scale bar, 50 μm.) (*B*) Representative [Ca^2+^]_i_ traces out of a total of 72 and 40 islets correspondingly subjected to 10 h of incubation with T2DM (*Upper*) or ALS-T2DM serum (*Lower*), followed by perifusion with 3.3 and then 11.1 mM glucose (G), respectively. (*C* and *D*) Insulin secretion from islets treated with T2DM or ALS-T2DM serum for 10 h followed by exposure to 3.3 or 11.1 mM G for 30 min. *P < 0.05 vs. the T2DM/3.3 mM G group (*C*). Net insulin secretion induced by 11 mM G in T2DM and ALS-T2DM groups. **P < 0.01 vs. the T2DM group (*D*). Experiments were done with 6 T2DM and 6 ALS-T2DM sera in triplicate. (*E*) Insulin content in islets exposed to T2DM or ALS-T2DM serum for 10 h. *P < 0.05 vs. the T2DM group. Experiments were done with 4 T2DM and 3 ALS-T2DM sera in triplicate.

We characterized [Ca^2+^]_i_ dynamics in β cells situated within intact islets during glucose stimulation. As shown in photomicrographs of Fura-2-loaded mouse islets, ALS-T2DM serum treatment made islets become irregular and disintegrated (Fig. 5 *A*, *Lower*). In striking contrast, incubation with T2DM serum did not alter the morphology of islets that were intact with spherical shapes and smooth boundaries (Fig. 5 *A*, *Upper*). Indeed, ALS-T2DM serum-treated islets showed chaotic [Ca^2+^]_i_ dynamics manifested as a relatively steady increase in [Ca^2+^]_i_ with tiny amplitude oscillations in response to 11.1 mM glucose (Fig. 5 *B*, *Lower* and *SI Appendix*, Fig. S4, *Lower*). However, islets exposed to T2DM serum displayed a normal [Ca^2+^]_i_ profile, characterized by fast oscillations superimposed on slow oscillations, following stimulation with 11.1 mM glucose (Fig. 5 *B*, *Upper* and *SI Appendix*, Fig. S4, *Upper*). These results reveal that ALS-T2DM serum does indeed potently derange [Ca^2+^]_i_ handling in β cells.

The primary function of β cells is glucose-stimulated insulin secretion that crucially depends on Ca_V_ channel-mediated Ca^2+^ influx and complex [Ca^2+^]_i_ dynamics (12, 13, 24, 33–35). ALS-T2DM serum-induced defects in [Ca^2+^]_i_ dynamics should cause impaired glucose-stimulated insulin secretion. T2DM serum- and ALS-T2DM serum-treated islets released a similar amount of insulin following incubation with 11.1 mM glucose (Fig. 5 *C* and *D*). However, insulin secreted from T2DM serum-treated islets at 11.1 mM glucose was significantly greater than that at 3.3 mM glucose, whereas insulin released from ALS-T2DM serum-treated islets at 11.1 mM glucose did not significantly differ from that at 3.3 mM glucose due to increased basal insulin release (Fig. 5 *C* and *D*). In addition, the insulin content of ALS-T2DM serum-treated islets was significantly lower than that of islets exposed to T2DM serum (Fig. 5*E*). These data suggest that exposure to ALS-T2DM serum interferes with the ability of the β cell to maintain adequate insulin release.

### Positive ALS-T2DM Sera Reduce Mouse Islet Cell Viability in an IgG- and Ca_V_1 Channel-Dependent Manner.

**Fig. 6.**
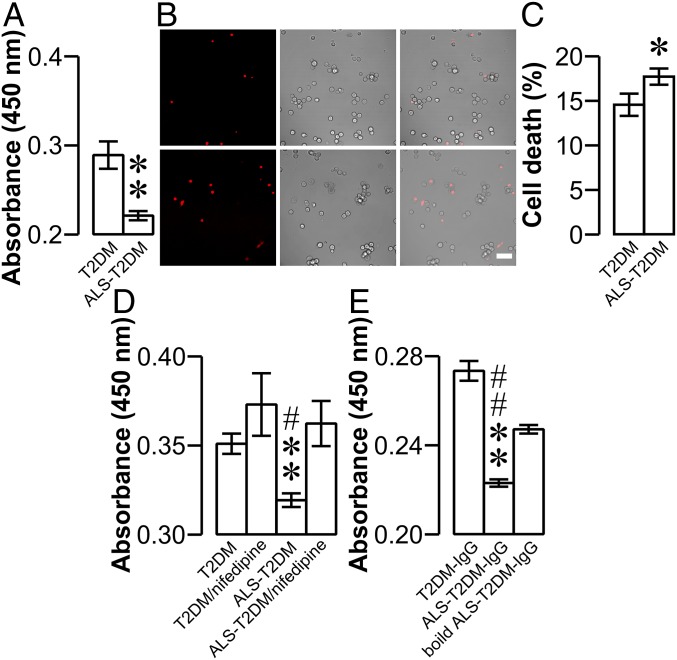
Effects of positive ALS-T2DM sera on mouse islet cell viability. (*A*) Mean WST-1 absorbance showing the viability of dissociated islet cells exposed to T2DM or ALS-T2DM serum. Experiments were done with 5 T2DM and 7 ALS-T2DM sera. **P < 0.01 vs. the T2DM group. (*B*) Example SYTOX Orange fluorescence (*Left*), transmitted light (*Middle*), and their overlay images (*Right*) of dissociated islet cells incubated with T2DM (*Upper*) and ALS-T2DM serum (*Lower*) followed by exposure to SYTOX Orange nucleic acid stain. (Scale bar, 50 μm.) (*C*) Mean percentage of dead cells labeled with SYTOX Orange nucleic acid stain in T2DM and ALS-T2DM groups. Experiments were done with 3 T2DM and 3 ALS-T2DM sera in triplicate. *P < 0.05 vs. the T2DM group. (*D*) Mean WST-1 absorbance showing the viability of dissociated islet cells exposed to 3 T2DM sera or 3 ALS-T2DM sera in the absence and presence of the Ca_V_1 channel blocker nifedipine. **P < 0.01 vs. the T2DM group and ^#^*P* < 0.05 vs. the ALS-T2DM/nifedipine group. (*E*) Mean WST-1 absorbance in islet cells following 24 h exposure to T2DM-IgG, ALS-T2DM-IgG, or boiled ALS-T2DM-IgG. Experiments were done with 3 T2DM-IgGs, 3 ALS-T2DM-IgGs, or 3 boiled ALS-T2DM-IgGs. **P < 0.01 vs. the T2DM-IgG group and ^##^*P* < 0.01 vs. the boiled ALS-T2DM-IgG group.

The exaggerated Ca_V_1 channel-mediated Ca^2+^ influx, increased [Ca^2+^]_i_, and disturbed [Ca^2+^]_i_ dynamics in islet cells exposed to ALS-T2DM serum might explain the destructive action of this serum on islet integrity and islet insulin content. Therefore, we examined the possible effects of ALS-T2DM serum on islet cell survival. WST-1 assay showed that ALS-T2DM serum exposure significantly decreased islet cell viability, as reflected by significantly reduced WST-1 absorbance, in comparison to treatment with T2DM serum (Fig. 6*A*). Furthermore, cell death imaging with SYTOX Orange nucleic acid stain revealed that SYTOX Orange–positive profiles, representing dead nuclei, were significantly greater in dissociated islet cells incubated with ALS-T2DM serum compared to T2DM serum-treated ones (Fig. 6 *B* and *C*). Furthermore, the selective Ca_V_1 channel blocker nifedipine fully ablated ALS-T2DM serum-induced reduction of islet cell viability (Fig. 6*D*). In addition, treatment with ALS-T2DM-IgGs significantly reduced mouse islet cell viability in comparison to incubation with T2DM-IgGs. The effects of ALS-T2DM-IgG on islet cell viability were effectively ablated by boiling (Fig. 6*E*). The results demonstrate that positive ALS-T2DM sera interfere with islet cell survival in an IgG- and Ca_V_1 channel-dependent manner. Taken together, our results suggest that ALS-T2DM serum treatment destroys islet cells by excessively increasing Ca_V_1 channel-mediated Ca^2+^ influx and [Ca^2+^]_i_ and then pathologically translating the exaggerated [Ca^2+^]_i_ to eventual mitochondrial dysfunction.

## Discussion

We have identified a subgroup of ALS-T2DM patients who have positive sera that exaggerate [Ca^2+^]_i_ in pancreatic islet cells upon depolarization. This suggests that these positive sera are likely to interfere with Ca_V_ channels via serum molecular constituent(s). Indeed, we demonstrate that pathogenic IgG is accommodated in the sera of this subgroup of ALS-T2DM patients. This not only establishes a mechanistic link between ALS and T2DM but also suggests a potential role of altered humoral immunity in the development of ALS-associated T2DM.

Importantly, we reveal that ALS-T2DM serum significantly increases whole-cell Ca^2+^ currents predominantly passing through Ca_V_1.2 channels in mouse β cells (24). This mechanistically explains how ALS-T2DM serum and ALS-T2DM-IgG promote K^+^-evoked [Ca^2+^]_i_ responses and pinpoints that Ca_V_1.2 channels most likely serve as downstream targets of ALS-T2DM serum and ALS-T2DM-IgG. This finding is intriguing since ALS-T2DM serum is verified to functionally interfere with mouse β cell Ca_V_1.2 channels, which almost exclusively mediate the nifedipine-sensitive Ca^2+^ currents (11–13, 36, 37).

Interestingly, we found that ALS-T2DM-IgGs are strong enough to compete with an IgG rabbit polyclonal antibody specific to the extracellular epitope of Ca_V_α_2_δ1 subunits in living islet cells and in tsA-201 cells stably expressing Ca_V_α_2_δ1 subunits. This is in accordance with the fact that the Ca_V_α_2_δ1 subunits are entirely exposed to the extracellular space and thereby the most accessible to serum components among all β cell Ca_V_1.2 channel subunits. In addition, the Ca_V_α_2_δ1 subunit serves as an indispensable building element of the β cell Ca_V_1.2 channel complex to up-regulate the conductivity and surface expression of functional Ca_V_ channels (12, 25–27). Based on our results, we propose that ALS-T2DM-IgGs serve as autoantibodies that immunocapture Ca_V_α_2_δ1 subunits in the plasma membrane, thereby enhancing Ca_V_1 channel-mediated Ca^2+^ influx and [Ca^2+^]_i_ in islet cells. This process likely occurs through allosteric activation and/or gradual accumulation of Ca_V_1 channels in the β cell plasma membrane since antibodies in some cases activate and accumulate rather than inhibit and neutralize their binding partners (38, 39). This autoimmune mechanism is particularly interesting since ALS autoantibodies have been shown to target only Ca_V_1.1, Ca_V_2.1, and Ca_V_2.2 channels prior to the present work (2, 18, 20–23). Now the immunocapture of β cell Ca_V_α_2_δ1 subunits by ALS-T2DM-IgGs and consequent up-regulation of β cell Ca_V_1.2 channels come into the picture. Importantly, the present work reveals that humoral autoimmunity arises as the pathogenic machinery leading to this subset of diabetes. It is intriguing to explore if such a humoral mechanism not only operates in the pathogenesis of T2DM but also in that of type 1 diabetes in addition to a T cell–mediated autoimmune destruction of β cells (40). Moreover, our findings offer a causal link between ALS and T2DM and shed light on potential therapeutic targets for prevention and treatment of a subgroup of ALS-T2DM patients.

The mitochondrion is a vulnerable target downstream of excessive accumulation of [Ca^2+^]_i_ to mediate Ca^2+^-dependent impairments in cell function and viability (30, 41). Consequently, islet cells exposed to positive ALS-T2DM sera not only display exaggerated [Ca^2+^]_i_ but also mitochondrial dysfunction. These findings give a strong rationale for the ALS-T2DM serum-induced islet cell dysfunction and death. We found that positive ALS-T2DM sera derange [Ca^2+^]_i_ dynamics, impair insulin secretion, and drive islet cell death in a Ca_V_1 channel- and IgG-dependent manner. The occurrence of these pathological phenotypes is well accounted for by impaired mitochondrial function. Our data thus suggest that IgG autoantibodies in ALS-T2DM sera immunocapture Ca_V_α_2_δ1 subunits in the plasma membrane, resulting in a destructive exaggeration of [Ca^2+^]_i_ followed by its pathological translation into mitochondrial dysfunction, subsequent impairment of insulin secretion, eventual islet cell death, and diabetes. Of note, in vivo ALS-T2DM-IgGs may target peripheral insulin-sensitive tissues of patients, leading to insulin resistance (8).

The exact reason why only a fraction of ALS patients develop diabetes is unclear but is most likely due to a heterogeneous β cell sensitivity to the evoked [Ca^2+^]_i_ challenges. In addition, both ALS and T2DM represent an etiologically heterogeneous group of disorders where patients are likely to experience particular environmental challenges on top of their specific genetic predisposition. In this context, a specific combination of genetic and environmental factors may trigger the production of autoantibodies to the autoantigen Ca_V_α_2_δ1 subunit. This leads to a pathological Ca_V_ channel conductivity with resulting damage to motor neurons and islet cells.

In conclusion, the present work demonstrates that a subgroup of ALS-T2DM patients have sera that enhance Ca_V_1 channel-mediated Ca^2+^ influx, resulting in exaggerated [Ca^2+^]_i_. These effects are attributed to the fact that the sera accommodate cytotoxic IgG autoantibodies that immunocapture Ca_V_α_2_δ1 subunits. As a consequence, impairments in [Ca^2+^]_i_ dynamics, mitochondrial function, insulin secretion, and cell viability occur. This suggests that cytotoxic ALS-T2DM-IgG autoantibodies serve as a causal link between ALS and T2DM by interacting with and modulating the Ca_V_α_2_δ1–Ca_V_1 channel complex, which may lay the foundation for a pharmacological treatment strategy for patients suffering from a combination of these severe diseases.

## Methods

### Animals.

Male C57BL/6J mice aged from 8 to 10 wk were purchased from The Jackson Laboratory (Bar Harbor, ME). All animal experiments were conducted according to the guidelines of the Ethics Committee at Pohang University of Science and Technology (2011-0001) and the Animal Experiment Ethics Committee at Karolinska Institutet (N183/13).

### Enrollment of Patients.

Seventeen patients with ALS-T2DM (11 males and 6 females, age: 55.0 ± 2.1), 12 HSs, 9 patients with ALS, and 14 patients with T2DM (7 males and 7 females, age: 60.4 ± 2.5) were randomly selected (*SI Appendix*, Table S1). One ALS patient carrying a SOD1 mutation was excluded from the study (*SI Appendix*, Table S1). Ethical approval to use serum from patients was obtained from Hanyang University Hospital in Seoul, Korea (HYUH 2006-04-001-004). Blood samples were collected after obtaining written informed consent. Actual patients had no known family history of neuromuscular disease or wasting. All subjects diagnosed with T2DM were well controlled with hypoglycemic agents or insulin.

Additional experimental procedures are presented in the *SI Appendix*.

### Data Availability.

All of the data, associated protocols, code, and materials for this study are available within the paper and its *SI Appendix*.

## Supplementary Material

Supplementary File
