# Supplementary material for: IgGs from patients with amyotrophic lateral sclerosis and diabetes target CaVα2δ1 subunits impairing islet cell function and survival
Source: Proc Natl Acad Sci U S A. 2019 Dec 11;116(52):26816–22. doi: 10.1073/pnas.1911956116 (PMC6936400; doi:10.1073/pnas.1911956116)
Supplement: Supplementary File [file pnas.1911956116.sapp.pdf]

Supplementary Information for

IgGs from patients with amyotrophic lateral sclerosis and diabetes target $Ca_V\alpha_2\delta1$ subunits impairing islet cell function and survival

Yue Shi, Kyoung Sun Park, Seung Hyun Kim, Jia Yu, Kaixuan Zhao, Lina Yu, Ki Wook Oh, Kayoung Lee, Jaeyoon Kim, Kanchan Chaggar, Yuxin Li, Annette C. Dolphin, William A. Catterall, Sung Ho Ryu, Shao-Nian Yang and Per-Olof Berggren

Yue Shi
E-mail: yue.shi@ki.se
William A. Catterall
E-mail: wcatt@uw.edu
Shao-Nian Yang
E-mail: shao-nian.yang@ki.se
Per-Olof Berggren
E-mail: per-olof.berggren@ki.se

This PDF file includes:

Supplementary text
SI Methods
Figures S1 to S5
Tables S1
SI References

www.pnas.org/cgi/doi/10.1073/pnas.1911956116

Supplementary Information Text

SI Methods

Isolation and Cultivation of Pancreatic Islets and Islet Cells. Mouse pancreatic islets were isolated by standard collagenase digestion. Single islet cells were obtained by dispersing islets in Ca^{2+}-free medium with accutase (Gibco, Carlsbad, CA). Islets, single islet cells and RINm5F cells were seeded onto glass coverslips and cultured in RPMI 1640 medium supplemented with 10% fetal bovine serum, 2 mM L-glutamine, and 100 U/100 µg/ml penicillin/streptomycin (Invitrogen, Carlsbad, CA) at 37ºC in humidified atmosphere of 5% CO_2 in air (1, 2).

Generation and Cultivation of tsA-201 Cells Stably Expressing $Ca_V\alpha_2\delta1$ Subunits. Briefly, tsA-201 cells were stably transfected with $Ca_V\alpha_2\delta1$-pcDNA 3.1-zeo(+) vector and selected with 200 µg/ml zeocin (Invitrogen) to obtain the cells stably expressing $Ca_V\alpha_2\delta1$ subunits. The selected clones were grown in DMEM medium supplemented with 10% fetal bovine serum, 2 mM GlutaMAX and 100 U/100 µg/ml penicillin/streptomycin as well as 200 µg/ml zeocin (Invitrogen) at 37ºC in humidified atmosphere of 5% CO_2 in air. This approach is identical to that described in the previous work done by Davies *et al.* (3).

Serum Preparation and IgG Purification. Two batches of sera from 17 patients with ALS-T2DM as well as 12 HSs, 8 patients with ALS and 14 patients with T2DM as controls were collected in the early morning before they took any medicines, identically sterile-processed, and stored at -20°C until used. The sera were heat-inactivated by incubation at 56°C for 30 min. Cells were incubated overnight in RPMI 1640 culture medium supplemented with 10% of the actual serum. Serum IgG was purified using specific gel-based spin column purification (Melon Gel IgG purification resin; Pierce/Thermo Fisher Scientific). The IgG purification kit was based on a resin containing a proprietary ligand that only allows IgG to pass through. The column support was used to remove non-antibody serum proteins and isolate serum IgG. Briefly, 500 µl diluted serum was added to the mini-spin columns prepared according to the manufacturer's instructions, and IgG was eluted by centrifugation at 3000 *g*. Eluted fractions were concentrated by lyophilization and tested by sodium dodecyl sulfate polyacrylamide gel electrophoresis (SDS-PAGE) before use.

$[Ca^{2+}]_i$ Measurements. Islets, single islet cells and αTC1-6 cells (ATCC® CRL-2934; ATCC, Manassas, VA, USA) attached to glass coverslips were pretreated with the different sera, IgGs or other reagents and then loaded with 2 µM fura-2/AM for 30 min at 37°C in HEPES buffered solution containing (in mM) 125 NaCl, 5.9 KCl, 2.56 $CaCl_2$, 1.2 $MgCl_2$, 25 HEPES, and 3 glucose (pH 7.4) (4). A glucose concentration of 11.1 mM was used for islet stimulation. After loading, glass coverslips containing islets and cells were mounted into an open perifusion chamber and maintained at 37ºC. The Fura-2 F340/F380 ratio was measured to represent $[Ca^{2+}]_i$. The light source was equipped with a xenon arc lamp and an integrated shutter (Lambda DG-4, Sutter Instrument Company), and coupled to the microscope (Olympus ix 71) via a liquid light guide. The 16-bit grayscale images with a binning of 1 x 1 were captured every second (exposure time ranged from 100 to 300 ms) with a cooled EM-CCD camera (ImagEM X2, Hamamatsu). The camera and shutter were controlled by MetaFluor software (Molecular Devices). Data were analyzed by the same software. Cells with bright $[Ca^{2+}]_i$ signal defined the regions of interest (ROIs). ROI signals were calculated by subtracting background noise signal.

SYTOX Orange Staining and Confocal Microscopy. Cultured mouse islet cells on glass coverslips were incubated with 0.1 µM SYTOX Orange nucleic acid stain (Molecular Probes, Eugene, Oregon, USA) for 10 min. The stained cells were examined under a Leica TCS SP2 confocal laser scanner equipped with a 543-nm He-Ne laser and connected to a Leica DM IRBE microscope (Leica Microsystems Heidelberg GmbH) (2, 4). SYTOX orange was excited by a 543 nm laser line, and the resultant emission was captured using a Leica PL APO 40.0x/0.85 water objective at 550-610 nm.

Cell Membrane Fractionation, Immunoprecipitation, SDS-PAGE and Immunoblot Analysis. RINm5F cells were homogenized on ice in 500 µl of homogenization buffer containing 10 mM

tris(hydroxymethyl)aminomethane (TRIS), 10 mM EGTA, 10 mM EDTA, 320 mM D-sucrose, 1 mM PMSF and a protease inhibitor cocktail (Roche Diagnostics, Mannheim, Germany) (pH 7.4). The homogenate was centrifuged at 5000 *g* for 2 min at 4°C to remove cell debris and nuclei. The resultant samples were centrifuged in a Beckman TLA-100.2 rotor at 250,000 *g* and 4ºC for 30 min. The pellet was collected as crude membrane fraction and solubilized in immunoprecipitation buffer consisting of 10 mM TRIS, 150 mM NaCl, 10 mM EGTA, 10 mM EDTA, 1% triton X-100, 1 mM PMSF and a protease inhibitor cocktail (Roche Diagnostics, Mannheim, Germany) (pH 7.4) at 4ºC for 1 h. The solubilized membranes were subjected to centrifugation in a Beckman TLA-100.2 rotor at 250,000 *g* and 4ºC for 15 min and the supernatant was collected as detergent-soluble membrane proteins. 1000 µg of detergent-soluble membrane proteins were cleaned with 5 µg non-immune rabbit IgG (Merckmillipore) and 20 µl protein A/G PLUS-agarose beads (Santa Cruz Biotechnology). The cleaned samples were immunoprecipitated with 10 µg rabbit polyclonal antibodies to Ca_V1 subunits (Anti-$Ca_V1.2A$, produced in Dr. Catterall's laboratory), non-immune rabbit IgG (Merckmillipore), ALS-T2DM-IgG or T2DM-IgG together with 30 µl protein A/G PLUS-agarose beads (Santa Cruz Biotechnology). Thereafter, the resultant immunoprecipitates were denatured by heating at 56ºC for 20 min in SDS sample buffer and separated in discontinuous gels consisting of a 4% acrylamide stacking gel (pH 6.8) and a 8% acrylamide separating gel (pH 8.8). The separated proteins were then electroblotted to hydrophobic polyvinylidene difluoride membrane (Amersham Hybond P 0.45 PVDF; Amersham, Buckinghamshire, UK). The blots were blocked by incubation for 1 h with 5% non-fat milk powder in a washing buffer, containing 20 mM TRIS, 150 mM NaCl and 0.05% Tween 20 (pH 7.5), and then incubated overnight at 4ºC with rabbit polyclonal antibodies against $Ca_V1.2$ subunits (Anti-$Ca_V1.2B$, 1:200; Alomone Labs, Jerusalem, Israel), ALS-T2DM-IgG or T2DM-IgG. After washing, the blots were incubated with the secondary antibodies (horseradish peroxidase-conjugated goat anti-rabbit IgG; 1:50,000; Bio-Rad, Hercules, CA) at room temperature for 45 min. The immunoreactive bands were visualized on ChemiDoc™ Imaging System (Bio-Rad) with the Clarity™ Western ECL Substrate kit (Bio-Rad) (2, 4).

Plasma Membrane Fluorescence Labeling, Immunocytochemistry and Confocal Microscopy. Islet cells and tsA-201 cells stably expressing $Ca_V\alpha_2\delta1$ subunits, encoded by a rat $Ca_V\alpha_2\delta1$ cDNA (Accession number: M86621), adherent to glass coverslips were first labeled with rabbit polyclonal anti-$Ca_V\alpha_2\delta1$ antibodies (1:50; Catalogue number: ACC-015, Alomone Labs, Jerusalem, Israel), which recognize human, rat and mouse $Ca_V\alpha_2\delta1$, at 4ºC for 1 h and then incubated with a mixture of di-8-ANEPPS (25 µM; Invitrogen, Carlsbad, CA) and goat anti-rabbit IgG coupled to Alexa 633 (1:100; Molecular Probes) at 4 ºC for 1 h. The percentage sequence identity between human and rat, human and mouse and rat and mouse is 93.2%, 96.5% and 96.4%, respectively. Alexa 633 and di-8-ANEPPS were excited by 633 nm and 488 nm laser lines, respectively, and the resultant emissions were captured using a Leica PL APO 63x/1.20 water objective at 645-800 nm and 500-645 nm, respectively (2, 4).

Measurements of Mitochondrial Membrane Potential. Cultured mouse islet cells on glass coverslips were pretreated with T2DM and ALS-T2DM sera for 12 and 24 h, respectively. The pretreated cells were incubated in HEPES-buffered solution containing (in mM) 125 NaCl, 5.9 KCl, 2.56 $CaCl_2$, 1.2 $MgCl_2$, 25 HEPES, 3 glucose and 1 mg/ml bovine serum albumin (pH 7.4) at 37°C for 90 min. The cells were loaded with 32 µM rhodamine 123 for 10 min at 37 °C. The loaded rhodamine 123 was excited at 490 nm and the resultant emission was collected at 515-565 nm every 5 seconds. During the measurements, cells were stimulated with 11 mM glucose for 4 min and subsequently 10 µM carbonilcyanide p-triflouromethoxyphenylhydrazone (FCCP, Sigma-Aldrich).

Electrophysiological Recordings. Mouse islet cells were subjected to conventional whole-cell patch-clamp analysis with an EPC-10 patch clamp amplifier (HEKA Elektronik, Lambrecht/Pfalz, Germany) following different treatments. Cells were bathed in the external solution (in mM): 138 NaCl, 10 TEACl, 10 $CaCl_2$, 5.6 KCl, 1.2 $MgCl_2$, 5 HEPES, and 3 glucose (pH 7.4). The pipette solution contained (in mM) 150 N-methy-D-glucamine, 125 HCl, 10 EGTA, 1.2 $MgCl_2$, 3 Mg-ATP, and 5 HEPES (pH 7.15). Borosilicate glass electrodes (1.2 mm outside diameter, Warner

Instrument) were pulled with a vertical pipette puller (PC-10, Narishige), and had tip resistances ranging between 2 and 3 MΩ when filled with pipette solution. All recordings were performed at room temperature. The amplitude of whole-cell Ca^{2+} currents was normalized to cell capacitance. Acquisition and analysis of data were done using Patchmaster (HEKA Elektronik) (5, 6). Most reagents were purchased from Sigma-Aldrich. Nifedipine was purchased from Tocris.

Insulin Secretion Assay. Measurements of glucose-induced insulin secretion were performed with isolated islets subjected to overnight culture. Ten islets were pooled in batches and incubated at 37°C for 30 min in a HEPES buffered solution (pH 7.4) containing (in mM): 125 NaCl, 5.9 KCl, 1.2 $MgCl_2$, 2.56 $CaCl_2$, and 3 or 11 glucose supplemented with BSA at a concentration of 1 mg/ml. Supernatants were carefully aspirated and static insulin secretion from islets was assayed using the mouse insulin ELISA kit (ALPCO) (4).

WST-1 Assay. Cell viability was assessed under the different experimental conditions using the PreMix Water-Soluble Tetrazolium Salt (WST-1) Cell Proliferation Assay System (Takara Bio Inc.). Cells were seeded in a 96-well microplate and cultivated for 12 and 24 hours in RPMI 1640 medium containing different sera (10%) and purified IgGs (50 and 100 μg/ml), respectively. Subsequently, the culture medium was replaced with a 10:1 solution of the fresh culture medium and WST-1 reagent. The microplate was incubated for 4 hours at 37°C and then metabolically active viable cells were quantified by measuring absorbance at 450 nm in a microplate reader. Incubation with 100 μg/ml IgGs for 24 h was optimal for assessing effects of IgGs on cell viability (*SI Appendix,* Fig. S5).

Statistical Analysis. All data are presented as mean ± SEM. Statistical significance was determined by Student's *t* test, one-way ANOVA followed by least significant difference (LSD) test and two-way ANOVA followed by the Bonferroni post-test. The significance level was set to less than 0.05.

SI Figures

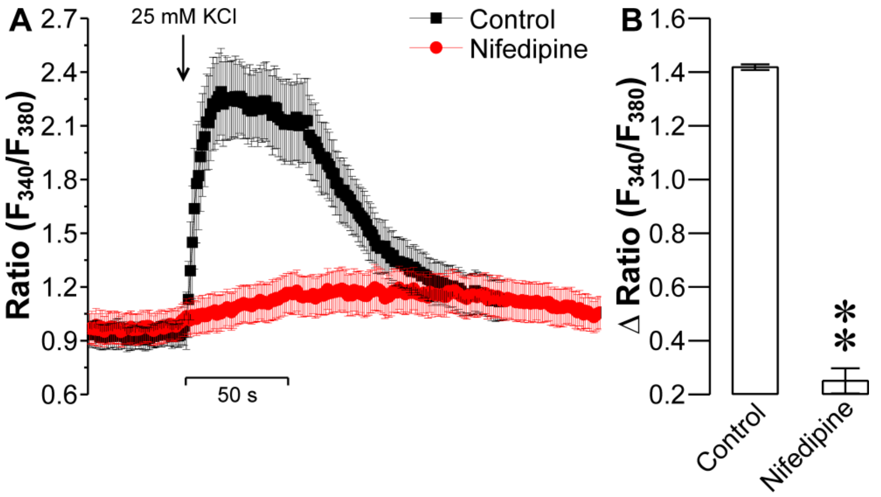

Fig. S1. Effects of the selective CaV1 channel blocker nifedipine on K^+-induced $[Ca^{2+}]_i$ responses in mouse islet cells. (*A*) K^+-induced $[Ca^{2+}]_i$ responses in control mouse islet cells and those pre-treated with nifedipine. (*B*) Delta fura-2 F340/F380 ratios showing net increase in $[Ca^{2+}]_i$ induced by K^+ depolarization in control mouse islet cells (120 cells) and those pre-treated with nifedipine (224 cells). $^{**}P < 0.01$ *vs.* control group.

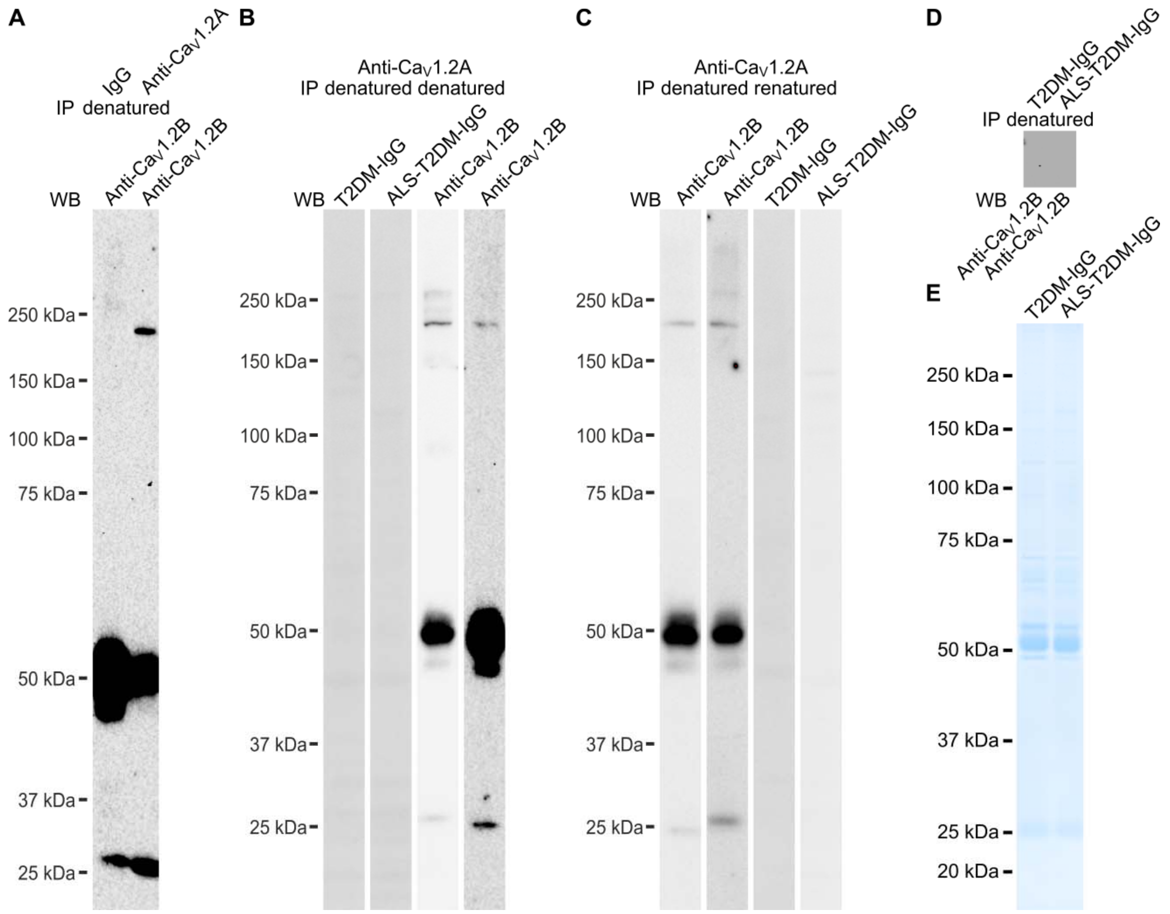

Fig. S2. Detection of association between ALS-T2DM-IgGs and Ca$_V$1.2 subunits in RINm5f cell membrane fractions. (*A*) Immunoreactive bands of Ca$_V$1.2 subunits not fished out by nonimmunne IgG (left lane), but pulled down by anti-Ca$_V$1.2 antibody A and recognized by anti-Ca$_V$1.2 antibody B (right lane). IP: immunoprecipitation. WB: Western blot. (*B*) Immuneprecipitated Ca$_V$1.2 subunits undetected by ALS-T2DM-IgGs or T2DM-IgGs, but recognized by anti-Ca$_V$1.2 antibody B under denaturing conditions. (*C*) Immuneprecipitated Ca$_V$1.2 subunits recognized by anti-Ca$_V$1.2 antibody B under denaturing conditions, but undetected by ALS-T2DM-IgGs or T2DM-IgGs under renaturing conditions. (*D*) No Ca$_V$1.2 subunits pulled down by ALS-T2DM-IgGs or T2DM-IgGs. (*E*) No additional specific proteins fished out by ALS-T2DM-IgGs in comparison to T2DM-IgGs.

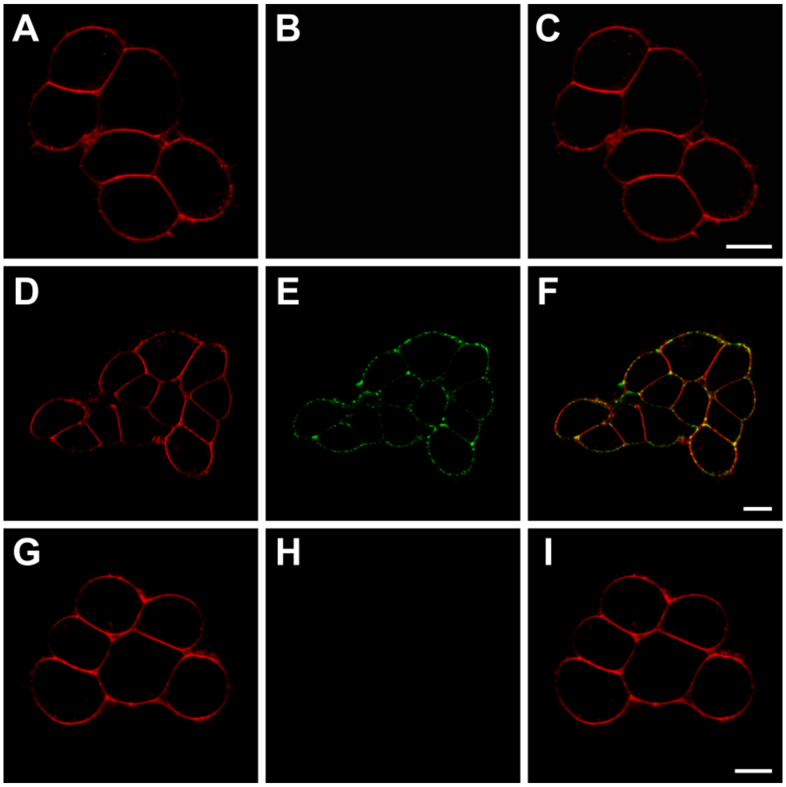

Fig. S3. Ca$_V\alpha_2\delta$1-specific immunofluorescence in the plasma membrane of living tsA-201 cells stably expressing Ca$_V\alpha_2\delta$1 subunits. (*A-I*) Representative di-8-ANEPPS fluorescence (*A*, *D*, and *G*), Ca$_V\alpha_2\delta$1 immunofluorescence (*B*, *E*, and *H*) and their overlay images (*C*, *F*, and *I*) of living control tsA-201 cells incubated with rabbit polyclonal anti-Ca$_V\alpha_2\delta$1 antibodies (*A-C*) and tsA-201 cells stably expressing Ca$_V\alpha_2\delta$1 subunits incubated with rabbit polyclonal anti-Ca$_V\alpha_2\delta$1 antibodies in the absence (*D-F*) and presence of the control peptide antigen (*G-I*). Scale bar = 10 μm.

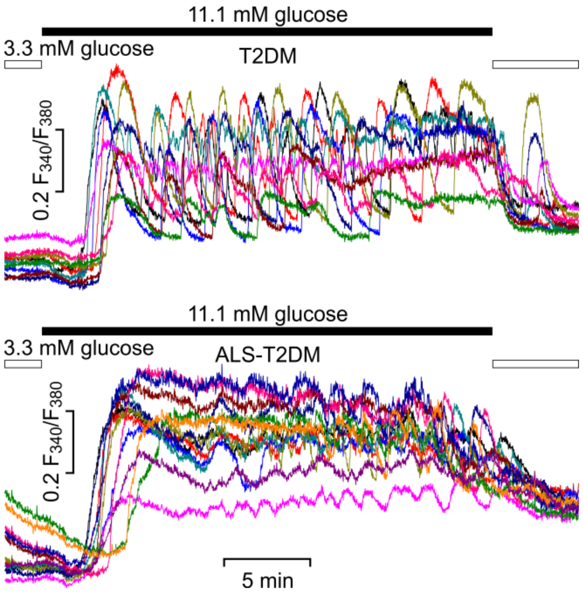

Fig. S4. Effects of positive ALS-T2DM sera on mouse islet $[Ca^{2+}]_i$ dynamics. $[Ca^{2+}]_i$ traces acquired during perifusion with 3.3 mM and 11.1 mM glucose in T2DM (upper panel) and ALS-T2DM serum-treated mouse islets (lower panel).

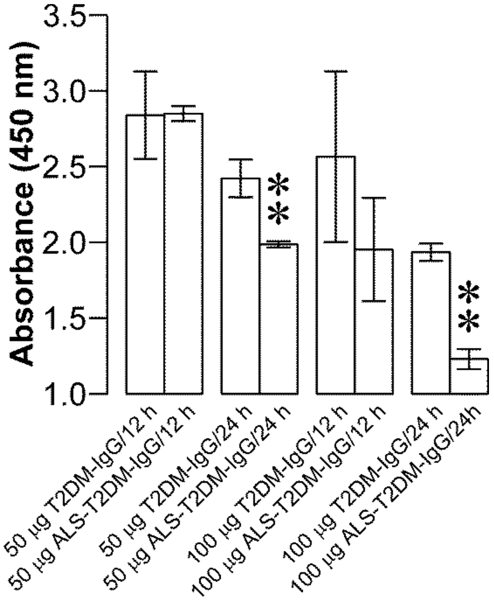

Fig. S5. Effects of IgG purified from positive ALS-T2DM sera on viability of αTC1-6 cells. Averaged WST-1 absorbance, reflecting viability, in cells exposed to T2DM-IgG and ALS-T2DM-IgG for 12 and 24 h, respectively. Incubation for 12 h with ALS-T2DM-IgG was not sufficient to alter WST-1 absorbance in αTC1-6 cells in comparison with treatment with T2DM-IgG. Treatment for 24 h with 50 and 100 μg ALS-T2DM-IgG significantly deceased WST-1 absorbance compared to 24-h incubation with 50 and 100 μg T2DM-IgG, respectively. ***P* < 0.01 *vs.* 50 or 100 μg T2DM-IgG/24 h group.

Table S1. Characterization of healthy subjects, ALS, T2DM and ALS-T2DM patients

Group	Sex	Age of onset ALS/T2DM (years)	Age at sampling (years)	Disease duration ALS/T2DM (months/years)	Site of onset ALS	ALSFRS-R score	Revised El Escorial criteria	Family history ALS/T2DM	SOD1 mutation	FBG (mg/dl)	A1C (%)	Use of OHA (/day)	Use of insulin
$Healthy_1$	F		51							110	NA	No	No
$Healthy_2$	M		53							102	6.5	No	No
$Healthy_3$	F		82							112	6	No	No
$Healthy_4$	F		62							96	6.3	No	No
$Healthy_5$	F		70							87	5.5	No	No
$Healthy_6$	F		65							107	5.8	No	No
$Healthy_7$	M		40							92	5.8	No	No
$Healthy_8$	F		50							87	5.9	No	No
$Healthy_9$	F		76							82	5.7	No	No
$Healthy_{10}$	F		61							93	6.2	No	No
$Healthy_{11}$	F		72							96	6.3	No	No
$Healthy_{12}$	M		60							88	6.1	No	No
ALS_1	M	48/	52	43/	Bulbar	14	Probable	No/Yes	Not detected	101	NA	No	No
ALS_2	M	39/	42	38/	Upper limb	8	Clinical probable lab supported	No/No	Not detected	85	5.6	No	No

ALS_3	M	47/	51	47/	Lower limb	25	Clinical probable lab supported	No/No	Not detected	119	NA	No	No
ALS_4	F	52/	58	78/	Lower limb	22	Probable	No/No	Not detected	109	5.9	No	No
ALS_5	F	46/	47	13/	Lower limb	39	Probable	No/Yes	Not detected	104	NA	No	No
ALS_6	F	56/	58	20/	Upper limb	45	Clinical probable lab supported	No/No	Not detected	100	5.8	No	No
ALS_7	M	52/	53	16/	Lower limb	45	Probable	No/No	Not detected	91	6.1	No	No
ALS_8	M	55/	55	6/	Lower limb	42	Clinical probable lab supported	No/No	Not detected	107	5.5	No	No
ALS_9	F	56/	57	14/	Lower limb	38	Clinical probable lab supported	Yes/No	detected	95	NA		
$T2DM_1$	F	/55	58	/3				/No		101	5.8	Met 500 mg	No
$T2DM_2$	M	/35	42	/7				/No		90	6	Met 1000 mg	No
$T2DM_3$	F	/49	67	/18				/No		112	6.1	Glim 2 mg/Met 1000 mg	No
$T2DM_4$	F	/49	50	/1				/No		108	5.7	Met 500 mg	No
$T2DM_5$	F	/53	60	/3				/No		129	6.6	Met 1000 mg	No
$T2DM_6$	M	/49	49	/0				/No		77	5.2	Met 500 mg	No
$T2DM_7$	M	/59	69	/10				/No		170	7.3	Met 1500	No

												mg/Sit 50mg/Rep 1.25 mg	
T2DM$_{8}$	M	/45	68	/23				/No		226	8.9	Met 1000 mg/Rep 2 mg	Yes
T2DM$_{9}$	F	/43	57	/14				/Yes		114	6.8	Gem 50mg/Met 1000 mg	No
T2DM$_{10}$	F	/51	56	/3				/No		190	7.6	Glim 2 mg/Met 1000 mg/Sit 10mg	No
T2DM$_{11}$	M	/64	64	/0				/No		319	11.8	Met 500 mg	Yes
T2DM$_{12}$	M	/51	61	/10				/No		145	7.8	Met 1000 mg/Lin 5 mg	No
T2DM$_{13}$	M	/38	74	/36				/No		174	7.7	Met 1000 mg	No
T2DM$_{14}$	F	/65	71	/6				/No		73	6.4	No	Yes
ALS-T2DM$_{1}$	M	44/35	48	45/13	Upper limb	16	Probable with lab support	No/No	Not detected	86	5.4	Glim 2 mg/Met 500 mg	No
ALS-T2DM$_{2}$	M	45/45	45	4/0	Bulbar	46	Probable with lab support	No/No	Not detected	140	6.6	Vog 0.4 mg	No
ALS-T2DM$_{3}$	F	53/45	55	20/10	Lower limb	31	Probable	No/No	Not detected	139	6.8	Glim 2 mg/Met 1000	No

												mg/Aca 100 mg	
ALS-T2DM$_4$	F	51/52	52	15/0	Lower limb	29	Probable	No/No	Not detected	105	5.4	Met 500 mg	No
ALS-T2DM$_5$	M	56/56	57	10/1	Bulbar	15	Definite	No/No	Not detected	114	6.3	Met 500 mg	No
ALS-T2DM$_6$	M	62/44	64	30/20	Upper limb	29	Probable	No/No	Not detected	137	5.7	Met 500 mg/Glib 2.5 mg	No
ALS-T2DM$_7$	M	41/43	43	18/0	Upper limb	31	Probable	No/No	Not detected	109	5.3	Met 500 mg	No
ALS-T2DM$_8$	M	48/47	49	12/2	Lower limb	45	Probable with lab support	No/No	Not detected	125	5.9	Aca 300 mg/Glic 30 mg	No
ALS-T2DM$_9$	M	58/60	60	15/0	Bulbar	34	Probable	No/No	Not detected	91	5.9	Met 500 mg	No
ALS-T2DM$_{10}$	M	46/48	50	51/2	Upper limb	17	Definite	No/No	Not detected	100	5.9	Met 500 mg	No
ALS-T2DM$_{11}$	M	58/58	62	38/4	Upper limb	18	Probable with lab support	No/Yes	Not detected	114	5.4	Met 1000 mg	No
ALS-T2DM$_{12}$	F	47/45	48	19/3	Lower limb	32	Probable	No/No	Not detected	109	5.1	Met 500 mg	No
ALS-T2DM$_{13}$	F	76/75	78	17/3	Upper limb	42	Probable	No/No	Not detected	138	7.8	Glim 2 mg/Met 1000 mg/Lin 5 mg	No
ALS-	M	60/32	62	24/30	Upper	36	Probable	No/Yes	Not detected	191	6.8	Glim 4	Yes

$T2DM_{14}$					limb							mg/Met 1000 mg/Lin 5 mg	
ALS-$T2DM_{15}$	M	49/48	52	33/4	Upper limb	40	Probable	No/No	Not detected	144	6.8	Sax 5 mg/Met 500 mg	No
ALS-$T2DM_{16}$	F	51/55	59	96/4	Bulbar	0	Probable	No/No	Not detected	175	6.2	No	Yes
ALS-$T2DM_{17}$	F	48/51	51	36/0	Lower limb	3	Probable	No/No	Not detected	328	8.9	Met 1000 mg/Lin 5 mg	Yes

Disease duration was defined as time period from onset of ALS to sampling time. ALSFRS-R score was obtained at sampling time. Aca, acarbose; ALSFRS-R, revised ALS Functional Rating Scale; A1C, hemoglobin A1C; F, female; FBG, fasting blood glucose levels; Gem, gemigliptin; Glib, glibenclamide; Glic, gliclazide; Glim, glimepiride; lab, laboratory; Lin, linagliptin; M, male; Met, metformin hydrochloride; NA, no data available; OHA, oral hypoglycemic agents; Rep, repaglinide; Sax, saxagliptin; Sit, sitagliptin; Vog, voglibose.

References

1. S. N. Yang *et al.*, Syntaxin 1 interacts with the L_D subtype of voltage-gated Ca^{2+} channels in pancreatic β cells. *Proc. Natl. Acad. Sci. U.S.A.* 96, 10164-10169 (1999).
2. S. N. Yang *et al.*, Glucose recruits K_{ATP} channels via non-insulin-containing dense-core granules. *Cell Metab.* 6, 217-228 (2007).
3. A. Davies *et al.*, The calcium channel alpha2delta-2 subunit partitions with $Ca_V2.1$ into lipid rafts in cerebellum: implications for localization and function. *J. Neurosci.* 26, 8748-8757 (2006).
4. P. O. Berggren *et al.*, Removal of Ca^{2+} channel β_3 subunit enhances Ca^{2+} oscillation frequency and insulin exocytosis. *Cell* 119, 273-284 (2004).
5. G. Yang *et al.*, $Ca_V1.2$ and $Ca_V1.3$ channel hyperactivation in mouse islet β cells exposed to type 1 diabetic serum. *Cell. Mol. Life Sci.* 72, 1197-1207 (2015).
6. Y. Shi *et al.*, Apolipoprotein CIII hyperactivates β cell Ca_V1 channels through SR-BI/β1 integrin-dependent coactivation of PKA and Src. *Cell. Mol. Life Sci.* 71, 1289-1303 (2014).
